# Implementing academic detailing for breast cancer screening in underserved communities

**DOI:** 10.1186/1748-5908-2-43

**Published:** 2007-12-17

**Authors:** Sherri Sheinfeld Gorin, Alfred R Ashford, Rafael Lantigua, Manisha Desai, Andrea Troxel, Donald Gemson

**Affiliations:** 1 Department of Health and Behavior Studies, Columbia University, 525 W 120^th ^Street, New York, NY, USA; 2 Department of Epidemiology, Mailman School of Public Health, Columbia University, 722 W 168^th ^Street, New York, NY, USA; 3 Herbert Irving Comprehensive Cancer Center, 1130 St. Nicholas Avenue, New York, NY, USA; 4 Harlem Hospital Center, MLK Pavilion, New York, NY, USA; 5 College of Physicians and Surgeons, Columbia University, 600 W 168^th ^Street, New York, NY, USA; 6 Department of Biostatistics, Mailman School of Public Health, Columbia University, 722 W 168^th ^Street, New York, NY, USA; 7 Department of Biostatistics and Epidemiology, University of Pennsylvania, 632 Blockley Hall, Philadelphia, PA, USA; 8 Author deceased, May 31, 2007

## Abstract

**Background:**

African American and Hispanic women, such as those living in the northern Manhattan and the South Bronx neighborhoods of New York City, are generally underserved with regard to breast cancer prevention and screening practices, even though they are more likely to die of breast cancer than are other women. Primary care physicians (PCPs) are critical for the recommendation of breast cancer screening to their patients. Academic detailing is a promising strategy for improving PCP performance in recommending breast cancer screening, yet little is known about the effects of academic detailing on breast cancer screening among physicians who practice in medically underserved areas. We assessed the effectiveness of an enhanced, multi-component academic detailing intervention in increasing recommendations for breast cancer screening within a sample of community-based urban physicians.

**Methods:**

Two medically underserved communities were matched and randomized to intervention and control arms. Ninety-four primary care community (*i.e*., not hospital based) physicians in northern Manhattan were compared to 74 physicians in the South Bronx neighborhoods of the New York City metropolitan area. Intervention participants received enhanced physician-directed academic detailing, using the American Cancer Society guidelines for the early detection of breast cancer. Control group physicians received no intervention. We conducted interviews to measure primary care physicians' self-reported recommendation of mammography and Clinical Breast Examination (CBE), and whether PCPs taught women how to perform breast self examination (BSE).

**Results:**

Using multivariate analyses, we found a statistically significant intervention effect on the recommendation of CBE to women patients age 40 and over; mammography and breast self examination reports increased across both arms from baseline to follow-up, according to physician self-report. At post-test, physician involvement in additional educational programs, enhanced self-efficacy in counseling for prevention, the routine use of chart reminders, computer- rather than paper-based prompting and tracking approaches, printed patient education materials, performance targets for mammography, and increased involvement of nursing and other office staff were associated with increased screening.

**Conclusion:**

We found some evidence of improvement in breast cancer screening practices due to enhanced academic detailing among primary care physicians practicing in urban underserved communities.

## Background

With targeted investments to improve access, breast cancer screening has reached near-parity between African Americans and whites; Hispanics still lag behind [1-3]. Breast cancer screening is not yet population-wide, however, as recommended by Healthy People 2010, and communities vary considerably in their screening rates [4]. These remaining disparities in screening contribute in part to the higher death rates from the disease among African Americans, Hispanics, American Indians/Alaskan Natives, and Asian Americans/Pacific Islanders as compared to white women, despite the highest incidence rates among white women [5]. Several recent meta-analyses and systematic reviews have highlighted the importance of physician recommendation to reducing these disparities [6-9].

Little is known about the breast cancer screening recommendation performance of physicians who practice in medically underserved areas, and few studies to improve such performance have been reported. Academic detailing has been found to be effective in many studies in which it has been evaluated [10,11], and represents a promising strategy for addressing the clinical and policy barriers to increasing physician breast cancer screening recommendations in medically underserved areas. Traditionally employed by pharmaceutical companies to promote prescription drug uptake among physicians, academic detailing entails a brief face-to-face intervention with the clinician, sometimes repeated at periodic intervals. When applied as part of a multi-component (enhanced) intervention, academic detailing is often supplemented with the dissemination of techniques and tools that address office-based barriers to screening [12,13]. It rests on constructs from well-established theories to increase physician behavioral change [14], including the Theory of Planned Behavior [15] and Social Cognitive Theory [16].

The objective of this study was to assess the efficacy of enhanced academic detailing in increasing recommendations for breast cancer screening in a sample of community-based urban physicians as compared to physicians in a similar community. Results of this group randomized trial based on medical audit data have been reported previously [10]. This report presents study findings based on primary care physician self-report data.\Findings from physician surveys are frequently used to effect policy change [17], and to examine the impact of national initiatives [18], despite over-reporting relative to medical audits and patient surveys [19]. To date, there have been few reported studies using either physician self-report or medical audit data on academic detailing as a method for increasing adherence to evidence-based breast cancer screening guidelines among medically underserved African American and Hispanic populations. This study adds to our knowledge of the effectiveness of academic detailing among PCPs serving these populations.

## Methods

The subjects and methods of the study have been described in detail elsewhere [10]. Using US census data, we matched and randomized primary care physicians in the New York City neighborhoods of northern Manhattan and the South Bronx to the intervention condition (northern Manhattan), and the comparison arm (South Bronx). To identify physicians working in these communities, we collected licensing lists from New York State, directories from local hospitals, and names from our physician advisory board. We conducted windshield and foot surveys of these communities to identify any additional physicians' offices. Of approximately 642 physicians in these communities who were contacted by telephone to assess eligibility, 359 devoted at least 50% of their practice to primary care, were community-based (*i.e*., not hospital-based), and were not expecting to leave the area over the coming year, and thus met the study criteria. As is common in studies of organizations [20], we enrolled only the most senior fulltime (and thus the most influential) physician in the office. We enrolled 192 (53%) of these physicians at baseline with a verbal consent. Of these, 87% completed both a baseline and follow-up survey, yielding a final sample of 168 offices (94 intervention and 74 comparison). The study was approved by the Institutional Review Board of Columbia University.

The physician self-report measures have been described previously [10]. Physicians' estimates of breast cancer screening practices were based on binary responses (yes/no) to the following questions about mammography and clinical breast examination (CBE): whether the physician conducts or recommends the procedure; if yes, the frequency of those screenings for asymptomatic women age 40 to 49, and age 50 and over. We also asked one question about teaching breast self-examination (BSE). Physician socio-demographic and medical practice characteristics were also measured. At follow-up, we administered a 12-item subscale to measure the process of implementation for the enhanced academic detailing intervention, that is, the presence or absence of tools, systems, or approaches that support breast cancer screening (*e.g*., computerized systems for tracking and reminding patients about regular screening tests). The subscale was developed and tested in previous prevention research [21,22].

### Multi-component (enhanced) academic detailing intervention

Implementation of the intervention has been described previously [10]. Ninety-seven percent of the intervention physicians received four academic detailing visits (average, 9.25 minutes) with self-learning packets from two Master's level health educators that highlighted the American Cancer Society breast cancer screening recommendations for asymptomatic women, age 40 and over.

To increase efficient contact with the intervention physicians, visits were supplemented by six dinner seminars; 46% of the intervention physicians attended a seminar. We also disseminated a newsletter to decrease attrition; 86% of intervention participants found the newsletter relevant to their practice. Office-based breast cancer prevention materials (adapted from previous research [21,22]) were shared with the physician and other staff.

Differences at baseline by condition were tested via chi-squared analyses (or Mantel Haentzel *X*^2 ^for screening recommendations) or by an analysis of variance (ANOVA). Missing data for the practice measures (< 5%) were imputed by the researchers with the mean value. When applicable, all p-values resulted from the use of two-sided tests.

## Results

The characteristics of physicians at baseline have been described elsewhere [10]. Few statistically significant differences were uncovered between participating physicians by arm. Both intervention and control groups increased their routine recommendation of mammography to asymptomatic women aged 50 and older (p = 0.05) and aged 40 to 49 (p = 0.02) from baseline to follow-up (see Table 1). The rates at post-test were nearly identical. There were statistically significant intervention effects from baseline to follow-up on increased CBE recommendations to women aged 50 and older (p < 0.0001) and those aged 40 to 49 (p = 0.002) relative to the comparison groups. The comparison group evidenced diminished screening behavior from baseline to follow-up, contributing to the intervention effect.

**Table 1:** Comparison of physician self-report of breast cancer screening recommendation practices by intervention and comparison groups (N = 168)^a^

	Intervention		Comparison		
	%	%	%	%	p-value^b^
	Baseline	Follow-up	Baseline	Follow-up	
Recommend mammography					
Age 40–49^c^	89	97	85	96	0.02
> age 50^c^	87	99	88	99	0.05
Recommend Clinical Breast					
Examinations (CBE)					
Age 40–49^c^	71	93	99	85	0.002
> age 50^c^	79	93	99	93	< 0.0001
Teach breast self-exam^e^	81	94	96	97	< 0.0001

^a^N = 168 (N = 94, intervention, N = 74, comparison)

^b^Two sided tests comparing post test scores by arm, with baseline scores as a covariate.

^c^Within the past two years

While the intervention physicians displayed a larger improvement in their teaching of BSE to women post-intervention (p < 0.0001), their overall rates were statistically equivalent to those of the comparison physicians (94% versus 97%).

Overall, at post-test, 77% of intervention physicians found the educational materials and approaches somewhat or very helpful to them; 59% reported using intervention-delivered physician or patient education materials that they had not used previously. Table 2 lists the uptake of the specific office-based intervention components at post-test. This study was designed to assess the effect of the omnibus intervention, not of any particular component. By documenting the uptake of specific components of the intervention, however, we can provide a clearer picture of the types of support that are most salient to this physician population.

**Table 2:** Primary care physician rates of preventive service-related practicess^a ^implemented via enhanced academic detailing intervention (N = 168)

	Intervention	Comparison	
	M (SD)%	M (SD)%	p-value^b^
**Acquiring information^c^**			
Participating in seminars or conferences on breast cancer detection	7.	0	0.002

**Physician self-efficacy^d^**			
Confidence that counseling patients about health behavior and lifestyle to result in their successfully modifying their behaviors	1.96 (0.82)	2.71 (0.88)	< 0.0001

**Office-based tools and techniques^e^**			
Using lists or flow sheets in patients' charts	33	34	0.01
Using card files or other paper tickler systems	14	17	0.05
Using notices or stickers on patients' charts	20	8	0.02
Using computerized tracking or prompting services	6	0.6	0.02
Reminder notices given or mailed to patients	26	16	0.16
Patient-held mini-records of preventive services	10	10	0.98
Performance targets for mammography^c^	52	8	0.009
Performance targets for clinical breast exams	44	6	0.57

**Patient Education^e^**			
Using pamphlets or other printed materials	44	41	0.03
Using wall posters or other graphic displays	41	38	0.04
Using video or slide presentations	6	8	0.26
Health risk appraisal instruments	7	4	0.80

**Nursing or other office staff and the delivery of preventive services^e^**			
Involving nursing or other office staff in tracking and prompting preventive care	18	7	0.03
Involving nursing or other office staff in counseling patients about preventive services	19	5	0.001

^a^Collected only at follow-up

^b^Two-sided tests comparing post test scores by arm using X^2^.

^c^Percent of participants who report "yes."

^d^Likert scale from 1–4, 1 = very confident 4 = not at all confident

^e^Percent use routinely

Physician acquisition of additional information on breast cancer detection was significantly greater in the intervention than in the comparison group at post-test (p = 0.002). Similarly, physician self efficacy in counseling for preventive behaviors was significantly higher in the intervention group than the comparison group at post-test (p < 0.0001; see Table 2).

Looking at office-based tools and techniques, chart prompts (via notices and stickers) were used more routinely by intervention physicians than by control physicians at post-test (p = 0.02), as were overall computerized tracking or prompting systems (p = 0.02). By contrast, paper-based lists and flow sheets in patients' charts (p = 0.01) and card files or other paper tickler systems (p = 0.05) were used more routinely in control offices than in intervention sites at post-test. At post-test, more than one-half (52%) of the intervention physicians worked in settings with routine use of performance targets for mammography, compared to 8% of the control physicians (p = 0.009). Fifty percent of the physicians had performance targets for clinical breast examinations; the rates across both arms were similar at post-test. The routine use of reminder notices given or mailed to patients and patient hand-held mini-records of preventive services were similar in intervention and comparison arms at post-test.

Printed pamphlets and other patient education materials (p = 0.03), wall, or other graphic displays (p = 0.04) were more common in intervention offices at post-test than in comparison sites; however, videos or slide presentations for patient education were similarly uncommon across both arms. The use of health risk appraisal instruments was uncommon (11% overall), and similar across both arms at post-test. The routine involvement of nursing and other office staff in tracking, prompting, and counseling patients about preventive services was more frequent in intervention than in control offices at post-test (p = 0.03, tracking and prompting; p = 0.001, counseling).

## Discussion

Multi-component enhanced academic detailing increased primary care physicians' recommendations for CBE among women age 40 and older relative to a comparison group. These findings are consistent with medical audit results from the patients of participating physicians (generalized linear mixed model analysis of medical record audit; OR = 2.13, 95% CI = 1.31, 3.46, p = 0.002) [10]. The consistency of the results on increasing CBE screening using different measures and across several studies suggests robust findings [10,23]. In addition, academic detailing is a moderate cost intervention – approximately $721.77 per participant [24]- by comparison to another physician-based screening intervention [25], increasing its feasibility in low-resource settings.

While physician recommendations for mammography and BSE increased over time among intervention vs. control physicians, there were improvements across both arms. The improvements among control PCP's in BSE performance were very slight and not statistically significant. Medical audit findings of patient data did demonstrate an intervention effect for mammography, however [10].

In a previous study of these physician cohorts [10], the overall number of preventive services that were implemented across both arms was similar at post-test. Nationally, only about one-half (49–54%) of primary care physicians have access to any data on their own practices, such as lists of patients by age group, diagnosis, or process-of-care or clinical outcomes data; only 15% of these data are generated internally [26,27]. Looking at the specific components in this study, however, we found more nuance. Physician involvement in additional educational programs, most often sponsored by a local academic medical center and its affiliates, and increased self-efficacy in counseling for prevention, chart reminders, as well as the use of computer-rather than paper-based prompting and tracking approaches, and the increased involvement of nursing and other office staff, were associated with increased screening recommendations. These findings are consistent with national data on support for implementing and maintaining high quality screening programs [28-30]. Given the low prevalence of in-office automated programs overall, the study findings further suggest that many offices in under-resourced communities do not yet have the organizational structures or processes necessary to support comprehensive office system re-design efforts that depend on information technology. In these settings, however, academic detailing enhances the physician's office management skills so that the practice is more organized toward prevention.

The sampling process used in this study allowed us to obtain a more accurate and comprehensive listing of local physicians than is generally found using nationwide lists such as the American Medical Association Master File. Further, we obtained relatively high rates of physician study participation (comparable to [31] and higher than the 21% enrollment obtained among health plan-affiliated provider organizations in [32]). The rate of completion of academic detailing in the study was 97%, the highest in any community-based intervention of this type yet reported (42%, [33]; 85%, [34]; 76%, [35]). These sets of findings suggest that the intervention can reach and engage geographically diverse physicians who serve medically underserved populations.

A feasibility study of academic detailing, using fewer visits (two) than in our study, found either group or individual contact acceptable [36]. In separate analyses, we found no differences in breast cancer screening recommendations between intervention physicians who also attended the seminars and those who did not, suggesting that additional contact in groups may not be necessary to effect behavior change.

As to limitations of the study, as stated earlier, the findings reflected physician over-reporting of their behaviors relative to medical audits [10], and population-based surveys [1-4,37,38]. The study's self-report findings should be interpreted with further caution, as the baseline levels of breast cancer screening were high, leading to a possible ceiling effect. Significant unmeasured differences between intervention and control groups at baseline and regression to the mean represent additional plausible explanations. While study participation may have sensitized comparison physicians to breast cancer screening, it is more likely that advocacy groups active during the intervention period [39] and national controversies, including the evidence both in support of and contesting routine breast cancer screening for women age 40 and older [40] influenced both groups.

Further studies, using other systematic measures of outcome, are necessary to confirm these findings. Both the applicability of the intervention to other cancer prevention and screening behaviors by primary care physicians, as well as the sustainability of the intervention over time are fruitful future research aims.

## Conclusion

The study suggests that enhanced academic detailing may be an effective implementation model for increasing evidence-based breast cancer screening recommendations among practices in urban areas of higher breast cancer mortality.
